# Postural control in gymnasts: anisotropic fractal scaling reveals proprioceptive reintegration in vestibular perturbation

**DOI:** 10.3389/fnetp.2024.1393171

**Published:** 2024-04-18

**Authors:** Madhur Mangalam, Ivan Seleznov, Elena Kolosova, Anton Popov, Damian G. Kelty-Stephen, Ken Kiyono

**Affiliations:** ^1^ Division of Biomechanics and Research Development, Department of Biomechanics, and Center for Research in Human Movement Variability, University of Nebraska at Omaha, Omaha, NE, United States; ^2^ Graduate School of Engineering Science, Osaka University, Osaka, Japan; ^3^ National University of Ukraine on Physical Education and Sport, Scientific Research Institute, Kyiv, Ukraine; ^4^ Department of Movement Physiology, Bogomoletz Institute of Physiology, Kyiv, Ukraine; ^5^ Department of Electronic Engineering, Igor Sikorsky Kyiv Polytechnic Institute, Kyiv, Ukraine; ^6^ Faculty of Applied Sciences, Ukrainian Catholic University, Lviv, Ukraine; ^7^ Department of Psychology, State University of New York at New Paltz, New Paltz, NY, United States

**Keywords:** fall, fractal, hurst exponent, motor disorder, multiscale, oriented fractal scaling component analysis, postural control

## Abstract

Dexterous postural control subtly complements movement variability with sensory correlations at many scales. The expressive poise of gymnasts exemplifies this lyrical punctuation of release with constraint, from coarse grain to fine scales. Dexterous postural control upon a 2D support surface might collapse the variation of center of pressure (CoP) to a relatively 1D orientation—a direction often oriented towards the focal point of a visual task. Sensory corrections in dexterous postural control might manifest in temporal correlations, specifically as fractional Brownian motions whose differences are more and less correlated with fractional Gaussian noises (*fGn*s) with progressively larger and smaller Hurst exponent *H*. Traditional empirical work examines this arrangement of lower-dimensional compression of CoP along two orthogonal axes, anteroposterior (AP) and mediolateral (ML). Eyes-open and face-forward orientations cultivate greater variability along AP than ML axes, and the orthogonal distribution of spatial variability has so far gone hand in hand with an orthogonal distribution of *H*, for example, larger in AP and lower in ML. However, perturbing the orientation of task focus might destabilize the postural synergy away from its 1D distribution and homogenize the temporal correlations across the 2D support surface, resulting in narrower angles between the directions of the largest and smallest *H*. We used oriented fractal scaling component analysis (OFSCA) to investigate whether sensory corrections in postural control might thus become suborthogonal. OFSCA models raw 2D CoP trajectory by decomposing it in all directions along the 2D support surface and fits the directions with the largest and smallest *H*. We studied a sample of gymnasts in eyes-open and face-forward quiet posture, and results from OFSCA confirm that such posture exhibits the classic orthogonal distribution of temporal correlations. Head-turning resulted in a simultaneous decrease in this angle Δ*θ*, which promptly reversed once gymnasts reoriented their heads forward. However, when vision was absent, there was only a discernible negative trend in Δ*θ*, indicating a shift in the angle’s direction but not a statistically significant one. Thus, the narrowing of Δ*θ* may signify an adaptive strategy in postural control. The swift recovery of Δ*θ* upon returning to a forward-facing posture suggests that the temporary reduction is specific to head-turning and does not impose a lasting burden on postural control. Turning the head reduced the angle between these two orientations, facilitating the release of postural degrees of freedom towards a more uniform spread of the CoP across both dimensions of the support surface. The innovative aspect of this work is that it shows how fractality might serve as a control parameter of adaptive mechanisms of dexterous postural control.

## 1 Introduction

Gymnastics training exerts a profound and transformative impact on postural performance (Asseman et al., 2004; Asseman et al., 2008; Hrysomallis, 2011; Paillard, 2017; Paillard, 2019), molding individuals into adept conductors of equilibrium and control. The intricate maneuvers and dynamic routines of gymnastics necessitate a heightened integration of proprioceptive acuity, spatial precision, and sophisticated neuromuscular coordination. The navigation through flips, twists, and landings compels continuous physiological adaptation, fostering the development of a finely tuned postural control system. Notably, while the scientific discourse explicitly recognizes the contribution of diverse muscle groups and the progressive enhancement of core strength to the observed postural stability, some remaining unanswered scientific questions center on how specific elements within gymnastics training selectively impact diverse facets of postural control, the enduring effects of such training on injury mitigation, and the intricate interplay between gymnastic-induced postural adaptations and the aging process. An essential question in the present manuscript is how simple perturbations from routine daily activities prompt the postural skills cultivated in gymnastics training.

Gymnastics training fosters the development of dexterity, supporting adult-like postural coordination and control even in children (Busquets et al., 2021). Notably, gymnastics training significantly affects response latency, particularly for upper body muscles situated along the frontal aspect and precisely along the anteroposterior (AP) axis (Debu and Woollacott, 1988; Calavalle et al., 2008; Zemková and Kováčiková, 2023). Even young gymnasts improve their ability to use proprioceptive information for postural stability (Garcia et al., 2011). Expert gymnasts have a distinctive capacity to reintegrate proprioceptive input with other modalities, particularly vision, which sets them apart from non-gymnast athletes (Vuillerme et al., 2001b). The attentional demands associated with regulating postural sway increase with task difficulty, yet gymnasts exhibit a more negligible effect during unipedal stances (Vuillerme and Nougier, 2004). This effect holds even without visual information, indicating adeptness at using alternative compensatory sensory modalities (Vuillerme et al., 2001a). Furthermore, gymnasts showcase increased instability in the postural center of pressure (CoP) compared to head movements compared to their non-gymnast counterparts (Gautier et al., 2008).

Understanding how gymnasts coordinate these exquisite postural feats may depend on understanding how they coordinate their quiet postural sway across time. Motor variations and sensory corrections proceed in a finely tuned coordination across time, and empirical indicators of correlations in sway can reveal the underlying control processes. Examining the 2D CoP trajectory of an individual reveals that CoP fluctuation at one timepoint might correlate with CoP fluctuations at other times (Duarte and Zatsiorsky, 2000; Blaszczyk and Klonowski, 2001; Błaszczyk et al., 2014). Postural sway might be correlated across time, with a stronger correlation likely between two points closer together. As we consider longer timescales, there is more room for the sway to vary. So, temporal correlations of CoP fluctuations might decay. However, an important question is how slowly those temporal correlations decay between successive CoP fluctuations. For instance, a gradual adjustment in muscle posture from fatigue may mean that CoP fluctuations might have more random fluctuations with weak temporal correlations as the body enacts faster changes in posture. This slow, gradual decay of correlations across time can leave long-range traces of this correlation-time “memory” within the time series. When this decay of correlation follows a single power law, we call it “monofractality.” The traditional examination of human postural dynamics often characterizes the monofractality of the 2D CoP trajectory as fractional Brownian motion (fBm), wherein sample-to-sample displacements resemble fractional Gaussian noise (fGn) (Burdet and Rougier, 2007; Kuznetsov et al., 2013). Detrended Fluctuation Analysis (DFA) offers a compelling analytical method to estimate these monofractal temporal correlations between CoP fluctuations that vary across longer separations in time Peng et al. (1994, 1995). It estimates a Hurst exponent *H*_*fGn*_, indicating the strength of this power-law decay. Specifically, *H*_*fGn*_ relates with how the *SD*-like variations in CoP fluctuations grow across many timescales, encoding how the correlation among sequential fluctuations might decay slowly across longer separations in time. The *H*_*fGn*_ reveals the strength of persistent correlations (0.5 < *H*_*fGn*_ < 1.0; large values in the CoP fluctuation time series are typically followed by large values and *vice versa*) or anti-persistent correlations (0 < *H*_*fGn*_ < 0.5; large values in the time series are typically followed by small values and *vice versa*) in CoP fluctuations over time (Hurst, 1951; Eke et al., 2002).

The fractal structure has offered a robust empirical connection to postural control strategies in non-gymnasts (Blaszczyk and Klonowski, 2001; Gilfriche et al., 2018). The fractal nature of postural CoP fluctuations in healthy adults is robust to sensory perturbations, such as alterations in visual input (Stambolieva, 2011). Learning to rely on non-visual proprioceptive inputs can be essential in the adaptability and flexibility of athletic postural control mechanisms (Coubard et al., 2014; Picot et al., 2022). Discerning the fractal architecture of CoP fluctuations may allow us to discern the reliance on proprioception. For instance, athletes exhibit postural control in sports demanding relatively swift throwing capabilities (e.g., handball and tennis), showing pronounced temporal correlations in CoP fluctuations (Caballero et al., 2020; Caballero et al., 2021). Athletes with elevated CoP fractal dimensions along the AP axis show greater flexibility in making more sparing use of vision while finding their execution of intricate posture-motor tasks more so on proprioception (Borzucka et al., 2020; Wyatt et al., 2021). In experimentation using galvanic vestibular stimulation (GVS) to destabilize posture, temporal correlations in CoP correlate inversely with the likelihood of post-GVS falls (Van den Hoorn et al., 2018); gymnastics practice counteracts postural control disruption caused by GVS (Hopper et al., 2014; Maitre and Paillard, 2016), although these adaptations tend to be very specific and the transfer effect is limited (Asseman et al., 2004; Asseman et al., 2008; Paillard, 2017; Paillard, 2019). Various non-gymnast models have shown that fractal fluctuations are an essential support in the coordination of postural adaptation, for instance, for allowing the body to respond to a destabilizing postural perturbation or engage in a secondary, more visual perceptual task which might also destabilize posture (Furmanek et al., 2021; Ihlen et al., 2013; Jacobson et al., 2021; Kelty-Stephen et al., 2021b; Kelty-Stephen et al., 2021a; Kelty-Stephen et al., 2023; Mangalam and Kelty-Stephen, 2021a; Mangalam et al., 2020a; Mangalam et al., 2020b; Mangalam et al., 2021; Morales and Kolaczyk, 2002; Palatinus et al., 2014; Shimizu et al., 2002). The present work aims to understand how gymnastic training might transfer to quiet standing and to examine the fractal temporal correlations in postural response to perturbation of quiet standing.

The implication of fractal temporal correlations in control provides an exciting window into the network connectivity pervading physiological systems. For instance, a prevailing understanding of postural control presumes that individual physiological mechanisms (e.g., visual, proprioceptive, and vestibular) might be so independent as to, for example, rank their relative prominence (Fitzpatrick and McCloskey, 1994; Peterka and Benolken, 1995; Ivanenko et al., 1999; Horlings et al., 2009; Wiesmeier et al., 2015). An incautious reading of the preceding paragraph might leave the incorrect view that fractal temporal correlations can be another curious intervention that can act upon various, separate processes. We see the value of a targeted intervention. However, the sometimes difficult-to-grasp fact is that fractality is not necessarily a localizable mechanism. Certainly, we can envision that the exogenous application of fractal stimulation, localized to any specific component or modality of the movement system, might yield some benefits (Nozaki et al., 1999b; Nozaki et al., 1999a; Raffalt et al., 2021; Raffalt et al., 2023; Rizzo et al., 2020; Soma et al., 2003). However tempting though such interpretations can be, fractality itself might reflect endogenous interactions spanning a wide range of scales bustling and, indeed, chaotically boiling over with interactions across scales (Wagman and Miller, 2003; Turvey and Sheya, 2017; Profeta and Turvey, 2018). Hence, fractality might reflect the unfolding of network relationships, widely presumed to be neural or neuronal (Plenz and Thiagarajan, 2007; Kello, 2013). It is also as likely to reflect networks of non-neural physiological media no less important for behavior and more important than previously thought to the function of any neural network (Ingber, 2006; Bashan et al., 2012; Bartsch et al., 2015; Ivanov et al., 2016; Bogdan, 2019; Ivanov, 2021). A wealth of intriguing theoretical research on networks and their fractal or multifractal implications suggests that networks featuring numerous connections spanning multiple scales of activity can occasionally yield a singular fractal dimension (Bak et al., 1987; Bak et al., 1988), sometimes many fractal dimensions (Bonachela and Munoz, 2009; Bonachela et al., 2010), and sometimes only “power law-like” processes that implicate fractal and multifractality dimensionalities under structural constraints (Stephen et al., 2009; Xiao et al., 2021). These network approaches appear limited by how best to understand the growth of new nodes (Gorochowski et al., 2018) to close the gap between the theoretical model and idiosyncratic constraints of the empirical model systems. We intend our work as an empirical branch to complement all of this theoretical work in which we can measure physiological time series and, much like Bogdan (2019) suggested, read the estimated sets of fractal dimensions as revealing the network dynamics—not localized in one tissue but spanning multiple scales of functional activity Turvey and Fonseca (2014). This relatively empirical use of fractal geometries allows network modeling across body parts (Carver et al., 2017; Mangalam et al., 2020a; b; Stephen et al., 2012b).

**FIGURE 1:**
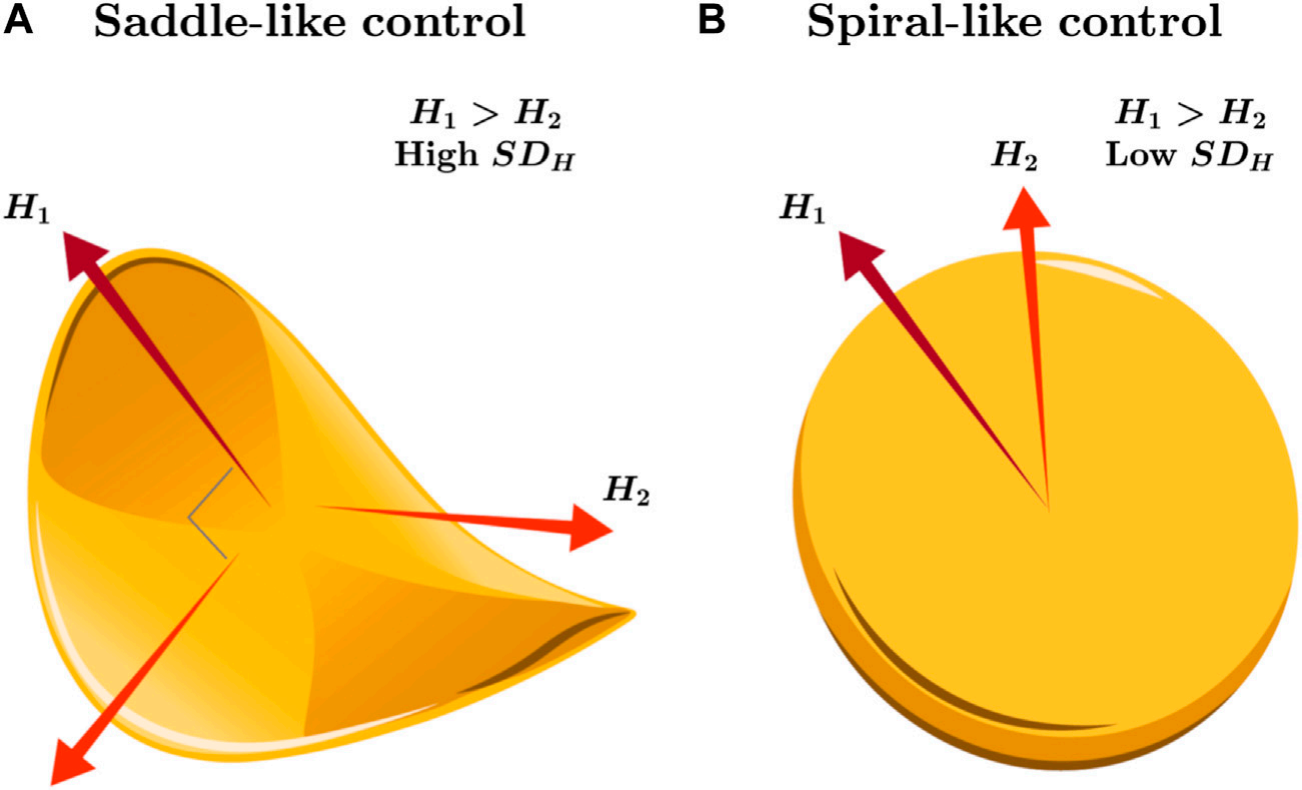
Schematic illustration of the saddle- and spiral-like strategies for controlling posture along the 2D support surface. **(A)** A stable equilibrium could emerge when examining temporal correlations predominantly in either the AP or ML axis but rarely in both, leading to a saddle-type control. For instance, heightened fractal scaling (i.e., large Hurst exponent *H*_1_) may indicate strong temporal correlations in postural sway along the AP direction, coupled with a reduced scaling exponent along the orthogonal ML direction (i.e., *H*_2_ < *H*_1_). This strategy could implicate more proactive control strategies along the AP axis and passive strategies along the ML axis with large spatial variability in temporal correlations (i.e., high *SD*_*H*_). **(B)** Alternatively, releasing the postural synergy constrained across the AP or ML axes in postural control might, for example, under perturbations or changes in orientation, disengage saddle-type control and pave the way for a transition to 2D spiral-type control, accompanied by relatively smaller spatial variability in temporal correlations (i.e., low *SD*_*H*_).

The 2D planarity of the support surface constitutes the widest possible constraints on postural control of the CoP trajectory. However, according to the tradition of movement science inspired by Bernstein (1967), coordination and control proceed by coalescing movement degrees of freedom into lower-dimensional solutions. So, the movement degrees of freedom may not always use the full dimensionality of the task constraints but may hover between collapsing and releasing degrees of freedom, fluidly reorganizing in response to unexpected perturbations. This point is as valid for postural control as for any other task (Latash et al., 2007; Latash, 2012). Models of postural variability make appeals to an intermittent controller sculpting distinctive sway patterns through a cyclical process, and by turns, this intermittent controller restrains CoP by collapsing its variability to a saddle-like stable manifold oriented along one “major” axis and, when it turns “off,” releases CoP into a spiraling circular expansion from the center point (Asai et al., 2009; Asai et al., 2013). That is, said another way, postural synergies compress variability from the two dimensions of the support surface to a relatively 1D axis of stability that avoids potentially less stable sway in the orthogonal direction. We often see this saddle of stability in a preponderance of temporal correlations in either the AP or ML axis but not both; for example, postural sway manifests augmented fractal scaling along the AP direction, accompanied by a diminished scaling exponent along the ML direction (Blaszczyk and Klonowski, 2001; Amoud et al., 2007; Kuznetsov et al., 2013), suggestive of more proactive and passive control strategies along the AP and ML axes, respectively (Figure 1A). Hence, in this traditional examination, the strategic allocation of temporal variability across the AP and ML axes by postural control mirrors its nuanced approach to more straightforward, task-oriented variability (Balasubramaniam et al., 2000). What we might expect, then, is that perturbations to posture might warrant the release of the ongoing postural synergy and consequent disengagement of saddle-type control, giving way to 2D spiral-type behavior (Figure 1B).

This saddle-type control of 2D CoP along AP and ML directions coincides with a conceptualization of postural control as an inverted pendulum fixed on two anatomical axes: the body executes rotations around one or more “hinges” aligned along the AP axis (Komura et al., 2005; Kuo, 2007; Kuo and Donelan, 2010). The saddle-type control is an apparent reason for finding temporal correlations that are strongest in one direction and weakest in the orthogonal direction, with stronger temporal correlations showing stronger signatures of coordination across time along one dimension than another. Despite the undeniable efficacy of measuring ground reaction forces along the AP and ML axes using force platforms, exclusive reliance on stability parameters within these orientations may inadvertently foster a misleading impression of their universal applicability across individuals (Granat et al., 1991; Sparto and Redfern, 2001). Indeed, effective postural control necessitates continuous integration of motor control spanning multiple directions (Peterka, 2002; Peterka and Loughlin, 2004; O’Connor and Kuo, 2009). In addition, the observed sway exhibits flexibility and fluidity, exceeding theoretical expectations from the inverted-pendulum model (Kelty-Stephen and Mangalam, 2023). Consequently, overfocus on orthogonal axes risks blinding us to spiral-type transients that might characterize the response to perturbation, allowing the formation of new saddle-type manifolds along novel task orientations.

Indeed, if the intermittent release of saddle-type control giving way to spiral-type control is adaptive, then the task-sensitive dissolution of orthogonality between the two postural-control directions could be a strategy for response to perturbation. Fortunately, Seleznov et al. (2020) have pioneered an analytical route that can help postural-control research to escape conventional AP-vs.-ML simplifications and explore how temporal correlations might transition between saddle and spiral orientations. This method, known as oriented fractal scaling component analysis (OFSCA), allows us to discern the directions, along the entire 2D support surface, of the strongest and weakest temporal correlations in sway. Although these directions can align with the anatomical AP and ML axes (Blaszczyk and Klonowski, 2001; Amoud et al., 2007; Kuznetsov et al., 2013), we know more about reorienting postural control from one direction to another (Balasubramaniam et al., 2000) and dramatically less about transient spiral-type states interspersed between postural adaptations. We might use OFSCA to analyze 2D CoP fluctuations to find saddle-type and spiral-type control signatures in the orthogonality or suborthogonality of the major and minor axes of temporal correlations in CoP.

The present study aims to clarify the foregoing possibilities. We seek here to call on the known postural dexterity of gymnasts to fill in empirical gaps and questions left from recent findings using the OFSCA. In recent comparisons of young healthy adults, older healthy adults, and adults with Parkinson’s disease (Mangalam et al., 2024b; Mangalam et al., 2024a), the healthy adults exhibited posture control with the strongest and weakest temporal correlations along two orthogonal directions, closely aligning with the anatomical AP and ML axes. Older adults show CoP with significantly narrower angles between the direction of the strongest and weakest temporal correlation, and adults with Parkinson’s disease showed dramatically more narrowing of this angle (Mangalam et al., 2024b). At first blush, this narrowing of directions of the strongest and weakest temporal correlations in CoP might look like the signature of a deficit. However, closer analysis suggests this narrowing might be an adaptive strategy: young healthy adults and older healthy adults showed significantly more narrowing of these two directions in experimental task settings that destabilized posture (e.g., closing eyes and standing on foam surface), and older adults with Parkinson’s disease showed no such change under the same task settings. Hence, healthy adults appear to exhibit brief narrowing of the axes in the CoP temporal correlations in response to perturbations. Meanwhile, individuals with Parkinson’s disease showed no flexibility in this narrowing (Mangalam et al., 2024b).

These OFSCA results may provide a novel empirical lens for testing the prior theoretical work [e.g., Asai et al. (2009, 2013)] emphasizing postural control vacillating intermittently between saddle- and spiral-type regimes. Younger adults show the clearest signature of this saddle-type control with their strongly orthogonal axes of the strongest and weakest temporal correlations, and older healthy adults may have less of this orthogonality in stable task settings. However, both show the narrowing of these directions that might correspond to greater 2D homogeneity in unstable conditions. The challenge for our interpretation is that individuals with Parkinson’s disease showed at once dramatically more narrowing and much less task-sensitive narrowing. A wider set of postural-control datasets of CoP, including healthy young and older adults alongside older adults with Parkinson’s disease, did show some task-sensitive narrowing of the two directions in response to unstable task settings (Mangalam et al., 2024a). But generally, it appeared that postural control in adults with Parkinson’s disease might spend disproportionately more time in the spiral-type mode, perhaps too much for a clear, unambiguous response to task manipulations. Is the loss of orthogonality a sign of disease? Or is it only a sign of disease when it shows weaker task sensitivity? So, we aimed to determine what orthogonality we might see in the 2D distribution of temporal correlations of gymnasts—under quiet standing with and without perturbations. Presumably, gymnasts show an adaptive response to the perturbation of quiet standing. So, we wish to use the gymnasts as a gold standard of quiet standing.

Our proposal to implicate the extremal axes of *H* in saddle-type and spiral-type regimes of control rests on traditions of control theory that are fundamentally topological. That is, invoking topological concerns “saddles” or “spirals” rests on an expectation of control strategies that are minimal and abstract and not directly invested in the anatomical constraints or details incidental to one or another model body or organism (Bernstein, 1967; Kelso, 1995; Holmes et al., 2006; Latash et al., 2007; Latash, 2012). Asai et al. (2009) did not invoke *H* as part of the control variables—they invoked the orthogonal dimensions of 1) the angle of the inverted pendulum sway and 2) the instantaneous change in the angle. Asai et al. (2009, 2013) did not even indicate whether they intended the angle to express rotation on the AP or the ML axis—AP and ML go entirely unmentioned. However, the actual observable of “angle” is not necessary to invoke this saddle/spiral control strategy, as it is replaceable with CoP or center of mass (CoM) (Nakamura et al., 2021). The angle of sway in any given axis is purely collinear with the movement of CoP in the same axis. As for the second axis, the convention of using instantaneous velocity reflects the convenient fact that the derivative of a pendular sine wave is the most straightforward algorithmic route to an orthogonal axis. A cosine wave is least correlated with the original sine wave. The proposed orthogonality of angular change from an angle matters more than the incidental fact of the pendulum, which is that the first derivative is orthogonal. If the angular position is collinear with AP or ML axes, we can replace angular change with the complementary axes (i.e., ML or AP, respectively). If angular change is orthogonal to angle, that is, a variable collinear with AP, then angular change must also be collinear with an axis orthogonal to AP—in this case, the topology on one (angular position vs. angular change) plane is the topology on the other (AP vs. ML). So, the details of which observables appear as labels are secondary to the topology, ideally a generic template for all anatomical/observable anchors.

We propose that the spatial distribution of sway variability presents a valuable perspective for discerning inter-individual variability in postural control, mainly influenced by gymnastics experience. We deliberately departed from conventional constraints: the strict adherence to AP versus ML directions and right angles. Instead, we sought a deeper understanding of postural control among trained gymnasts by examining task-specific alterations in the angles and directions between the smallest and largest fractal scaling patterns observed in 2D CoP trajectories. Our approach aimed to leverage the nuanced impact of gymnastics training on the robustness of postural control. We employed an innovative analytical technique known as oriented fractal scaling component analysis (OFSCA), as introduced by Seleznov et al. (2020) and subsequently used by Mangalam et al. (2024b), Mangalam et al. (2024a) to study postural deficits in older adults and individuals with Parkinson’s disease. This method decomposes the 2D CoP trajectories into directions corresponding to the most active and passive control mechanisms. This individualized portrayal of postural control was subsequently applied to the CoP 2D trajectories of a cohort of 17 trained gymnasts. Participants engaged in various conditions, including standing upright with eyes open versus closed and a perturbation condition involving rhythmic head rotation with both eyes open or closed. We intentionally selected this specific postural condition due to its inherent difficulty, anticipating it would yield the most pronounced angular anisotropy in postural sway. The chosen posture was anticipated to induce the most conspicuous directional variations in the individual’s swaying pattern. We posited that manipulating vision by closing the eyes and inducing vestibular perturbation through head rotation would each result in proprioceptive reintegration, evident in alterations to the spatial distribution of temporal correlations within CoP fluctuations.

Following the logic described above, we generated predictions with a twofold structure: predictions of effects for exogenous task manipulations (Hypothesis 1) and predictions of endogenous temporal correlations estimated by the OFSCA (Hypothesis 2). The OFSCA proceeds by estimating the angles *θ*_1_ and *θ*_2_ between the directions corresponding to the maximum and minimum values of the scaling exponent (i.e., indicated by *H*_1_ and *H*_2_ corresponding to the strongest and weakest temporal correlations, respectively) with respect to the force platform’s ML axis. It then returns the angle between these two axes as Δ*θ*. First, we expected that perturbing quiet standing with an instructed head-turning would narrow the angle between the two directions of the strongest and weakest temporal correlations (i.e., $\Delta \theta ={\hat{\theta }}_{1}∼{\hat{\theta }}_{2}$ ; Hypothesis 1a). We expected to replicate a similar effect for closing eyes and test for an interaction of closing eyes with head-turning (Hypothesis 1b). Second, we expected this angle to increase or decrease with the spatial variance of temporal correlations (Hypothesis 2a) and, concomitantly, to increase and decrease with the maximum and minimum values of the scaling exponent (i.e., *H*_1_ and *H*_2_, respectively) estimated by the OFSCA (Hypothesis 2b).

## 2 Materials and methods

### 2.1 Participants

A total of 17 gymnasts (11 men and 6 women; age: 19.7 ± 2.2 years; height: 174.0 ± 11.3 cm; body mass: 66.9 ± 13.4 kg) participated in this study. All participants exhibited high proficiency in their respective sports, having undergone at least 5 years of dedicated professional training. None of the subjects reported any musculoskeletal or neurological disorders that could impede their involvement in the study. Before their active participation, each athlete provided verbal and written informed consent.

### 2.2 Experimental protocol

We examined the spatial distribution of temporal correlations in the 2D CoP trajectory across four postural conditions.

• upright stance, eyes open, before head rotation (EO-B-HR)
• upright stance, eyes open, after head rotation (EO-A-HR)
• upright stance, eyes closed (EC-B-HR)
• head rotation counterclockwise, eyes closed (EC-HR)
• upright stance, eyes close, after head rotation (EC-A-HR)

Head rotation in both eyes open and eyes closed conditions were executed counterclockwise, following a self-selected, comfortable rhythm at approximately once per second, corresponding to ∼1 Hz. Hence, each participant performed a total of six postural trials, one trial for each condition. Each trial spanned 20 s, during which the postural CoP was recorded.

### 2.3 Data acquisition

Participants’ ground reaction forces in the six postural conditions were captured through a stabilographic force platform featuring four high-precision force transducers. The recording and subsequent analysis of stabilographic oscillations across four channels were executed through a NI6070E interface (National Instruments, Inc., Austin, TX) software scripted in LabView. The signals were sampled at a frequency of 100 Hz, and before input, all signals underwent band-pass filtering within the range of 0.5–200 Hz.

### 2.4 Oriented fractal scaling component analysis (OFSCA)

Comprehensively characterizing a 2D CoP trajectory typically relies on the consideration of two independent fractional Brownian motion (fBm) sample paths corresponding to CoP along the AP and ML directions, denoted as (*x* ^(1)^[*i*]) and (*x* ^(2)^[*i*]), where *i* = 1, 2, *…*, *N* denotes the trajectory’s length. This assumption posits that these two components consistently maintain orthogonality, implying that the scaling property of each angular component, specifically the projection onto a rotated direction, remains uniform and robust to any rotational transformation. However, this presumed spatial “isotropy” may not universally apply; instead, it might be an exception rather than the norm, as there is no inherent justification to assume that all natural 2D CoP trajectories inherently exhibit isotropic behavior (Qian et al., 1998; Jin et al., 2017). We employed the oriented fractal scaling component analysis (Seleznov et al., 2020) to delve into the spatially anisotropic autocorrelation characteristics of 2D CoP planar trajectories. This analytical approach assesses the angle-dependent scaling properties of the trajectory by utilizing a higher-order directional detrending moving average (DDMA) (Tsujimoto et al., 2016) and dissects the observed 2D CoP trajectory into two distinct components, each characterized by varying orientations and scaling properties.

**FIGURE 2:**
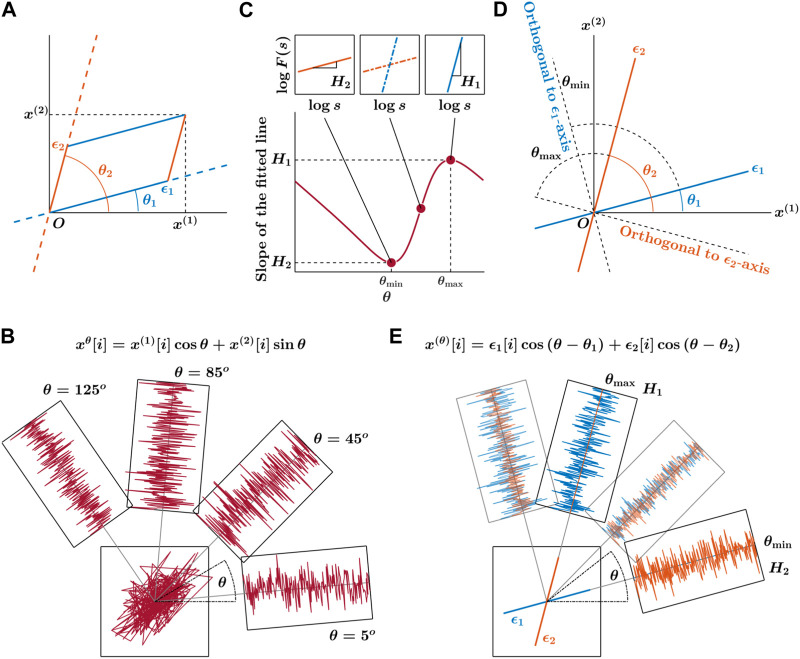
Primary depiction of the detection of angle-dependent temporally correlated components $\left\{\epsilon_{1}\left[i\right]\right\}$ and $\left\{\epsilon_{2}\left[i\right]\right\}$ of postural CoP in the $\left(x^{\left(1\right)},x^{\left(2\right)}\right)$ plane. The OFSCA concept asserts that the 2D CoP trajectory displays spatially distributed temporal correlations. The dominant directions, characterized by angles *θ*_1_ and *θ*_2_ relative to the horizontal reference, wield significant influence on posture control, evident in trajectories *ϵ*_1_ and *ϵ*_2_ **(A)**. Characterizing intrinsic patterns within these initial trajectories—originally treated as fractional Gaussian noise (fGn)—the OFSCA procedure kicks off by reshaping the observed 2D trajectory **(B)**. This transformation extends the trajectory across all angles within the 0 ≤ *θ* < *π* range. Subsequently, DDMA analysis gauges the strength of temporal correlations along these extended trajectories for each angle **(C)**. This framework pinpoints directions linked to the strongest and weakest temporal correlations, denoted as *H*_1_ and *H*_2_, corresponding to the major and minor axes of postural control, replacing the traditional AP and ML axes. Identifying the original components requires pinpointing where these scaling exponents reach their maximum and minimum values, labeled as *θ*_max_ and *θ*_min_. These values align perpendicular to the original component orientations **(D)**. Ultimately, the orientations of *H*_1_ and *H*_2_ are harnessed to reconstruct the authentic 2D CoP trajectory, encapsulating *ϵ*_1_ and *ϵ*_2_ **(E)**. Adapted from Seleznov et al. (2020) and reproduced from Mangalam et al. (2024b).

The OFSCA method characterizes the spatial distribution of temporal correlations within CoP fluctuations along the primary directions in which posture exhibits its extremal temporal correlations by analyzing the observed 2D CoP trajectories *ϵ*_1_ and *ϵ*_2_ at angles *θ*_1_ and *θ*_2_ relative to the horizontal reference direction (Figure 2A). It begins by estimating fractional Gaussian noise (fGn) within the observed 2D CoP trajectory, over all angles within the range 0 ≤ *θ* < *π* (Figure 2B). Subsequently, the DDMA analysis quantifies the strength of temporal correlations in these expanded trajectories at each angle. The original components indicate the directions *θ*_max_ and *θ*_min_ corresponding to angles between directions of the maximum and minimum values of these scaling exponents *H*_1_ and *H*_2_, respectively, from the force-plate ML axis (Figure 2C; these values consistently run orthogonal to the original orientations of the components, as shown in Figure 2D). Ultimately, the orientations of *H*_1_ and *H*_2_ allow reconstructing the actual 2D trajectory comprising *ϵ*_1_ and *ϵ*_2_, along with their corresponding directions (Figure 2E).

To gain a comprehensive understanding of the OFSCA method, we recommend the original work by Seleznov et al. (2020). Readers seeking additional insights into applying this method in clinical populations can refer to our earlier studies on individuals with Parkinson’s disease (Mangalam et al., 2024b, a).

### 2.5 Statistical analysis

We submitted each postural 2D CoP trajectory to the OFSCA and computed the angle Δ*θ* between the major and minor directions *θ*_1_ and *θ*_2_, respectively, of postural control as $\Delta \theta ={\hat{\theta }}_{1}∼{\hat{\theta }}_{2}$ . To clarify, the major and minor directions *θ*_1_ and *θ*_2_ indicate the directions of the components with the largest and smallest Hurst exponents *H*_1_ and *H*_2_, respectively. We used these same fixed-effect factors to model on Δ*θ* to test whether quiet standing exhibits more saddle-type organization of fractal correlations in one dimension of the support surface rather than the other (i.e., with larger Δ*θ*) and whether perturbing posture prompts a release of saddle-type constraints (i.e., exhibiting lower Δ*θ*). Then we elaborated our model with a fixed effect for *SD*_*H*_ to confirm that if greater Δ*θ* reflects the saddle-type orientation, it should correspond with greater *SD*_*H*_ and that. Conversely, the narrower Δ*θ* should reflect a more homogeneous, less variable spread of fractal correlations across both dimensions of the support surface. We included the random factor of participant identity by allowing the intercept to vary across participants. Statistical analyses were performed in R (R Core Team, 2013) using the package lme4 (Pinheiro et al., 2007). Significance was set at the two-tailed *α* level of 0.05.

## 3 Results

### 3.1 Gymnasts control posture along suborthogonal directions that deviate from the anatomical AP and ML directions

**FIGURE 3:**
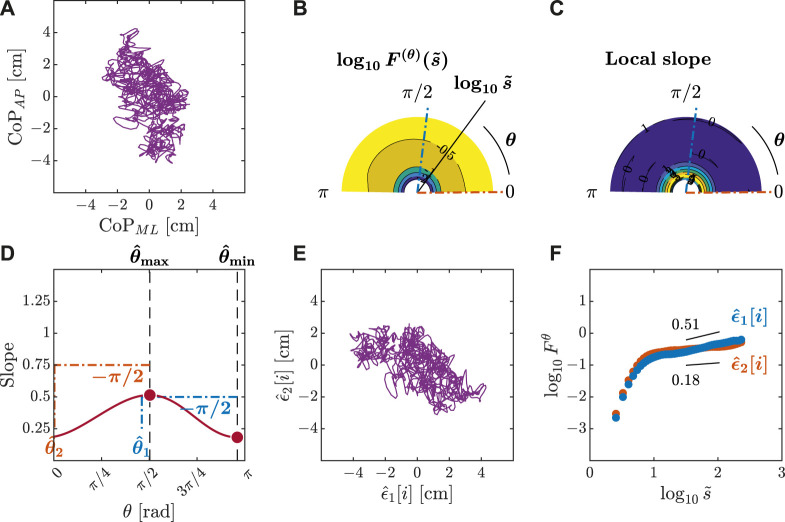
Orientation decomposition of the CoP trajectory of a representative gymnast with eyes closed before head rotation. **(A)** CoP along the anatomical AP and ML axes. **(B)** *θ*-dependent heterogeneity in CoP fluctuations, indicated by the angle dependence of $\log_{10}F^{\left(\theta \right)}\left(\tilde{s}\right)$ vs. $\log_{10}\tilde{s}$ , where $\tilde{s}∼s/2.74$ in the fourth order DDMA. **(C)** *θ*-dependence of the local slopes of $\log_{10}F^{\left(\theta \right)}\left(\tilde{s}\right)$ vs. $\log_{10}\tilde{s}$ , indicating the spatial distribution of temporal correlations. **(D)** *θ*-dependence of the slope in the range of $1.5<\log_{10}\tilde{s}<2$ . **(E)** Reconstructed CoP along the original directions of postural control, ${\hat{\epsilon }}_{1}\left[i\right],{\hat{\epsilon }}_{2}\left[i\right]$ . **(F)** Fluctuation functions of CoP along the original directions of postural control, ${\hat{\epsilon }}_{1}$ with ${\hat{\theta }}_{1}=83^{\text{o}}$ and ${\hat{\epsilon }}_{2}$ with ${\hat{\theta }}_{2}=1^{\text{o}}$ .

**FIGURE 4:**
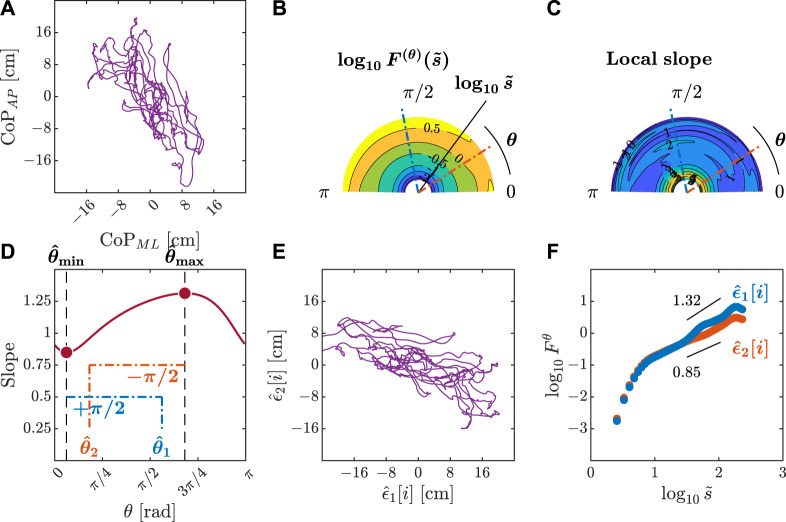
Orientation decomposition of the CoP trajectory of a representative gymnast with eyes closed during head rotation. **(A)** CoP along the anatomical AP and ML axes. **(B)** *θ*-dependent heterogeneity in CoP fluctuations, indicated by the angle dependence of $\log_{10}F^{\left(\theta \right)}\left(\tilde{s}\right)$ vs. $\log_{10}\tilde{s}$ , where $\tilde{s}∼s/2.74$ in the fourth order DDMA. **(C)** *θ*-dependence of the local slopes of $\log_{10}F^{\left(\theta \right)}\left(\tilde{s}\right)$ vs. $\log_{10}\tilde{s}$ , indicating the spatial distribution of temporal correlations. **(D)** *θ*-dependence of the slope in the range of $1.5<\log_{10}\tilde{s}<2$ . **(E)** Reconstructed CoP along the original directions of postural control, ${\hat{\epsilon }}_{1}\left[i\right],{\hat{\epsilon }}_{2}\left[i\right]$ . **(F)** Fluctuation functions of CoP along the original directions of postural control, ${\hat{\epsilon }}_{1}$ with ${\hat{\theta }}_{1}=101^{\text{o}}$ and ${\hat{\epsilon }}_{2}$ with ${\hat{\theta }}_{2}=32^{\text{o}}$ .

**FIGURE 5:**
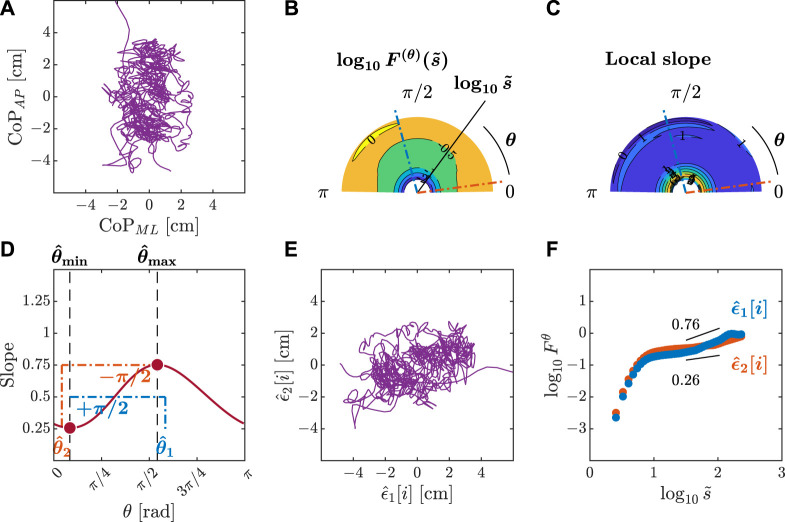
Orientation decomposition of the CoP trajectory of a representative gymnast with eyes closed after head rotation. **(A)** CoP along the anatomical AP and ML axes. **(B)** *θ*-dependent heterogeneity in CoP fluctuations, indicated by the angle dependence of $\log_{10}F^{\left(\theta \right)}\left(\tilde{s}\right)$ vs. $\log_{10}\tilde{s}$ , where $\tilde{s}∼s/2.74$ in the fourth order DDMA. **(C)** *θ*-dependence of the local slopes of $\log_{10}F^{\left(\theta \right)}\left(\tilde{s}\right)$ vs. $\log_{10}\tilde{s}$ , indicating the spatial distribution of temporal correlations. **(D)** *θ*-dependence of the slope in the range of $1.5<\log_{10}\tilde{s}<2$ . **(E)** Reconstructed CoP along the original directions of postural control, ${\hat{\epsilon }}_{1}\left[i\right],{\hat{\epsilon }}_{2}\left[i\right]$ . **(F)** Fluctuation functions of CoP along the original directions of postural control, ${\hat{\epsilon }}_{1}$ with ${\hat{\theta }}_{1}=105^{\text{o}}$ and ${\hat{\epsilon }}_{2}$ with ${\hat{\theta }}_{2}=8^{\text{o}}$ .

We submitted all postural CoP trajectories to the OFSCA with fourth-order DDMA. The plots depicted in Figures 3–5 represent the orientation decomposition of the 2D CoP trajectory of a representative gymnast in an upright stance, eyes closed, before head rotation (EC-B-HR), head rotation counterclockwise, eyes closed (EC-HR), and upright stance, eyes closed, after head rotation (EC-A-HR). We evaluated the angle dependence of $F^{\left(\theta \right)}\left(\tilde{s}\right)$ for the original CoP trajectory (Figures 3A, 4A, 5A) over the range of 0 ≤ *θ* < *π* in increments of *π*/179 rad, specifically indicating the spatial distribution of temporal correlations (Figures 3B, C, 4B). We set the scaling range $1.5<\log_{10}\tilde{s}<2$ (from 300 ms to 1 s) and estimated the slopes of linear regressions (Figures 3D, 4D, 5D) to find two representative orientations.

**FIGURE 6:**
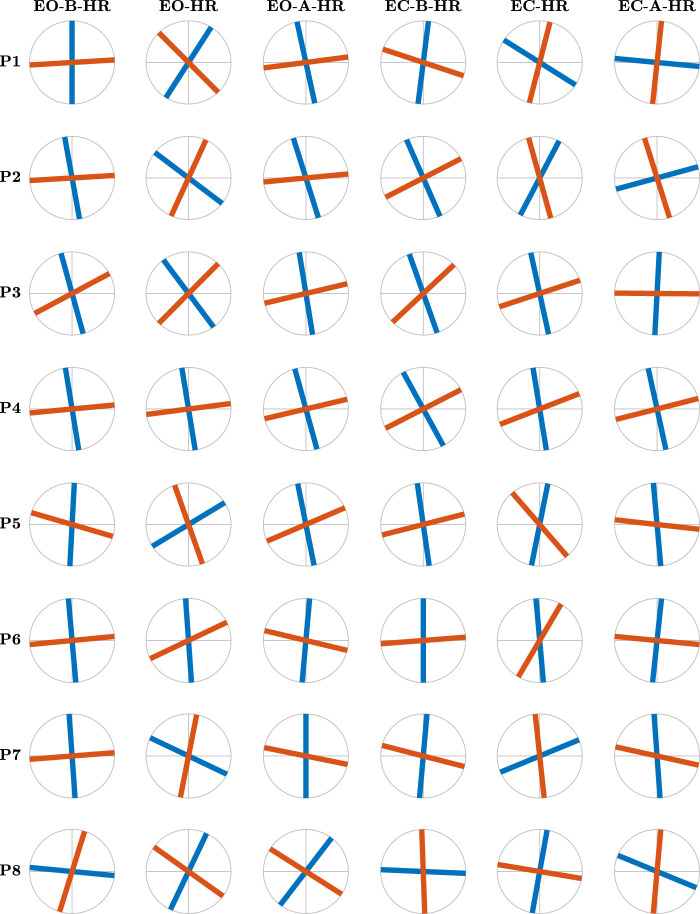
The major and minor directions of postural control, *θ*_1_ (*blue traces*) and *θ*_2_ (*red traces*), respectively, corresponding to the maximum and minimum strengths of temporal correlations in postural CoP, for participants 1–8 as revealed by the OFSCA. Notice that first, the two directions in which posture is controlled deviate from the anatomical AP and ML axes and that the angle between the two components is smaller than the 90^o^ between the AP and ML axes. Continued in Figure 7.

**FIGURE 7:**
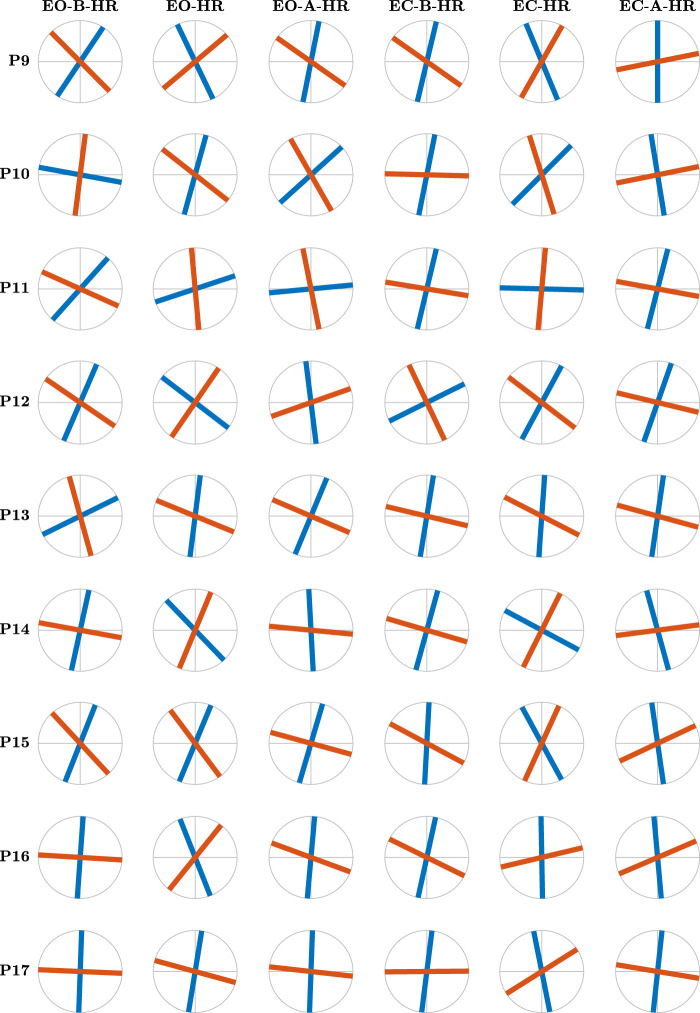
Figure 6 continues… The major and minor directions of postural control, *θ*_1_ (*blue traces*) and *θ*_2_ (*red traces*), respectively, corresponding to the maximum and minimum strengths of temporal correlations in postural CoP, for participants 9–17 as revealed by the OFSCA.

Contrary to the conventional belief attributing postural control to the anatomical AP and ML axes, the 17 gymnasts exhibited distinct maximum and minimum values of *H*_*fGn*_, *H*_1_ and *H*_2_, along divergent directions. In Figures 6, 7, we illustrate the major and minor directions of postural control—*θ*_1_ (*purple* traces) and *θ*_2_ (*yellow* traces)—corresponding to the maximum and minimum strengths of temporal correlations in postural CoP, *H*_1_ and *H*_2_, for each participant in each postural condition. Notably, these control directions deviate from the anatomical AP and ML axes, and the angle between them is often smaller than the 90^°^ between the AP and ML axes. Moreover, we observed significant variability in both angles among participants and across the six postural conditions for each participant. In Figure 6, the angles *θ*_1_ and *θ*_2_ exhibit a notable alignment with the AP and ML axes before head rotation with eyes open. However, during head rotation under the same condition, these angles significantly deviate from the AP and ML axes, only to realign, albeit to a lesser extent, post-rotation with eyes open. This pattern of variability is consistently observed across all three conditions for participant P5. Examining the variability in these directional control of posture across all participants reveals a low degree of variability before head rotation with eyes open (scan the left-most column in Figure 6 from top to bottom). However, participant variability is substantially increased during head rotation, particularly evident in the deviation from the anatomical AP and ML axes (scan the second-from-the-left column in Figure 6 from top to bottom). Participant P3, for instance, demonstrates a notably high degree of obliqueness in this context. Thus, we discovered that trained gymnasts manage posture along directions divergent from the anatomical AP and ML axes, actively altering these directions based on the demands of different task contexts.

### 3.2 Vision and head rotation differently affected the directions in which posture is controlled

The linear mixed-effect modeling generated coefficients *B* for each covariate, signifying the average change in Δ*θ* associated with group membership in class variable values or a unit increase in the corresponding continuous variable (Table 1). Each coefficient was accompanied by a standard error *SE*, representing the variation around the average change in Δ*θ*. We ran two models, the first to model only the exogenous factors indicating experimental task setting, namely, two class variables Vision (i.e., equaling “EyesClosed” versus a baseline value of “EyesOpen”) and Postural Condition (i.e., equaling “Head rotation” or “After” both relative to “Before”) and their interaction. We omitted this factor because the interaction failed to improve model fit [*χ* ^2^(2) = 2.045, *p* = 0.360]. Our second model added the endogenous factors *SD*_*H*_, *H*_1_, and *H*_2_ to test how the change from wider to narrower angle between the major and minor axes *θ*_1_ and *θ*_2_ correspond both to changes in spatial variability in axial temporal correlations (i.e., *SD*_*H*_) and to overall range (e.g., “max − min”) of temporal correlations. We present the estimated coefficients from the linear mixed-effect model in the format *B* ± *SE*, noting the corresponding *t*-statistic (equal to *B*/*SE*) and the *p*-value estimate based on Satterthwaite’s method.

**TABLE 1:** Outcomes of the linear mixed-effects (LME) models^a^ ^,^ ^b^ examining the influence of vision, postural conditions, and temporal correlations in CoP fluctuations on *θ*_1_, *θ*_2_, and Δ*θ*.

Predictor	*B* ± *SE*	*t*	*P* ^c^
Δ*θ*			
(Intercept)	81.204 ± 2.290	37.641	**2.000 × 10** ^**−15**^
Vision (EyesClosed ∼ EyesOpen)	−3.470 ± 1.876	−1.850	0.068
PosturalCondition (Head rotation ∼ Before)	−12.895 ± 2.298	−5.612	**2.460 × 10** ^**−7**^
PosturalCondition (After ∼ Before)	0.296 ± 2.298	0.129	0.898
Δ*θ*			
(Intercept)	76.469 ± 4.924	15.531	$<2.000\times 10^{-15}$
Vision (EyesClosed ∼ EyesOpen)	−1.863 ± 1.757	−1.060	0.292
PosturalCondition (Head rotation ∼ Before)	−12.229 ± 2.583	−4.735	7.570 × 10^−5^
PosturalCondition (After ∼ Before)	1.895 ± 2.129	0.890	0.376
*SD*_*H*_	690.160 ± 128.699	5.363	**5.440 × 10** ^**−6**^
*H*_1_	−221.185 ± 42.406	−5.216	**1.080 × 10** ^**−5**^
*H*_2_	225.479 ± 42.561	5.298	**7.420 × 10** ^**−6**^

^a^ Δ*θ* ∼Vision + PosturalCondition + (1|Participant).

^b^ Δ*θ* ∼Vision + PosturalCondition + *SD*_*H*_ + *H*_1_ + *H*_2_ + (1|Participant).

^c^ Boldfaced values indicate statistical significance at *p* < 0.05.

**FIGURE 8:**
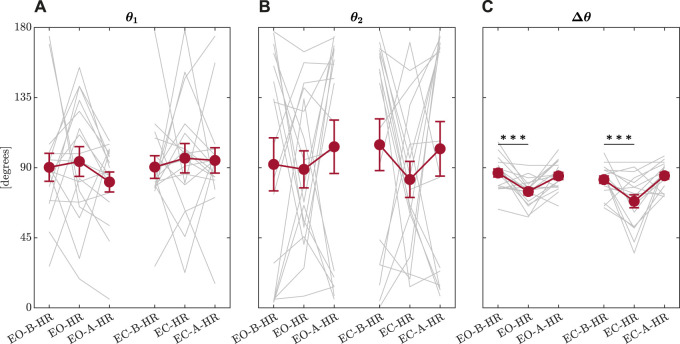
Summary of the output from the OFSCA. Mean values of *θ*_1_, the direction corresponding to the maximum value of temporal correlations, *H*_1_ **(A)**, *θ*_2_, the direction corresponding to the minimum value of temporal correlations, *H*_2_ **(B)**, and Δ*θ*, the angle between these two directions along which the posture is controlled **(C)**. Error bars indicate ± 1*S*.*E*.(*N* = 17). Light grey traces represent individual participants. *p < 0.05; ***p < 0.001.

The first model indicated that the head-turn perturbation had a much stronger effect of narrowing Δ*θ* than closing eyes, suggesting a more substantial support for Hypothesis 1a than Hypothesis 1b. The first model returned only trends in reduction of Δ*θ* by an average of 3.470 ± 1.876° (*t* = −1.850, *p* = 0.068; Figure 8). These values are consistent with previous findings in healthy young and older adults and individuals with Parkinson’s disease (Mangalam et al., 2024b; a). Head rotation during an upright stance showed considerably more reduction in Δ*θ* by an average of 12.895 ± 2.298 (*t* = −5.612, *p* = 2.460 × 10^−7^) with respect to the baseline trial before the head turn. Nonetheless, head rotation during an upright stance did not induce any discernible adaptation in the trial following head rotation, as Δ*θ* did not show any statistical difference with Δ*θ* observed in the baseline condition (*p* > 0.05). Thus, the reduction of Δ*θ* appears specific to the head turn and disappears as quickly as the gymnast returns to face-forward posture. This flexible rebound back to orthogonal axes thus makes the observed reduction of Δ*θ* appear less like liability and more like an adaptive response of postural control with minimal liabilities to the orthogonality of temporal correlations in subsequent postural sway.

The second model showed that incorporating information about endogenous temporal correlations dramatically improved our ability to predict the changes in Δ*θ* [*χ* ^2^(3) = 25.165, *p* = 1.426 × 10^−5^]. The effect of head-turning remained as strong as before, suggesting that the experimental manipulation was robust across many participants’ strategies of using temporal correlations to maintain postural control. Supporting the notion that orthogonality and generally greater Δ*θ* went hand in hand with the greater variability of presumably saddle-type control (Hypothesis 2a), there was a strong positive effect of *SD*_*H*_ on Δ*θ* (*B* ± *SE* = 690.160 ± 128.699, *t* = 5.363, *p* = 5.440 × 10^−6^), suggesting that greater variability of Hurst exponent encoding the strength of temporal correlations across the angular space went hand in hand with greater Δ*θ*. This finding helped confirm our intuition that greater angles between the major and minor axes closer to orthogonality might be associated with greater spatial variety as in saddle-type control (Hypothesis 2a). Conversely, this finding suggested that the narrower angles between the major and minor axes corresponded to more homogeneous values of *H* across all directions in the 2D plane. The second model indicated that the range between *H*_1_ and *H*_2_ might operate differently from the standard deviation. Specifically, although greater *SD*_*H*_ was associated with greater Δ*θ*, it appears that increasing *H*_1_ would decrease Δ*θ* (*B* ± *SE* = −221.185 ± 42.406°, *t* = −5.216, *p* = 1.180 × 10^−5^) and increasing *H*_2_ would increase Δ*θ* (*B* ± *SE* = 225.479 ± 42.561°, *t* = 5.298, *p* = 7.420 × 10^−6^). This evidence partially undermines our Hypothesis 2b that increasing the range of *H* would correspond to an increase of Δ*θ*: independently increasing either *H*_1_ or *H*_2_ while keeping the other constant would increase or decrease the range, respectively, but co-occur with smaller or larger Δ*θ*, also respectively. In short, although greater *SD*_*H*_ co-occurs with larger Δ*θ*, greater range co-occurs with smaller Δ*θ*. So, the effect of the extremal temporal correlations appears to operate in the opposite direction from the *SD* of temporal correlations. If we imagined a bell-shaped curve describing the distribution of angular temporal correlations, greater Δ*θ* corresponds to a fatter bell-shaped distribution with shorter tails. Conversely, smaller Δ*θ* corresponds to a thinner bell-shaped distribution with longer tails. Postural control appears to balance its spatial distribution of fractal temporal correlations between extremes and average variability.

## 4 Discussion

The present study tested two hypotheses about the nuanced spatial distribution of temporal correlations in CoP fluctuations among trained gymnasts. First, we predicted in Hypothesis 1a and 1b that head-turning and closing eyes would narrow the angle between the axes of the strongest and weakest temporal correlations in 2D CoP. Second, we predicted that greater Δ*θ* corresponding to the more orthogonal orientation of axes with the strongest and weakest temporal correlation would correspond to endogenous variability in temporal correlations—both in terms of the standard deviation of Hurst exponents *H*, encoded by *SD*_*H*_ (Hypothesis 2a) and in terms of the range of *H*, encoded by *H*_1_ and *H*_2_ (Hypothesis 2b). Results supported Hypothesis 1a but not Hypothesis 1b, and they supported Hypothesis 2a but not 2b. Regarding the first hypothesis, head-turning led to a concurrent reduction of the Δ*θ* that showed no signs of lingering once gymnasts returned their heads to a facing-forward position, but vision showed only a negative trend, indicating a change of Δ*θ* in negative direction but not a statistically reliable one. Hence, narrowing Δ*θ* might be an adaptive postural-control strategy that respects saddle-type postural synergy until a perturbation prompts the intermittent release of this synergy, giving way to a spiral-type mode. The rapid rebound of Δ*θ* with a return to face-forward posture suggests that the temporary reduction is specific to head-turning and not an ongoing burden to postural control.

We propose that the postural system’s capacity for brief, task-sensitive reductions in Δ*θ* may extend the body of fractal temporal-correlational evidence of intermittent postural control. In particular, we propose that the intermittent adoption of orthogonal and suborthogonal axes of extremal fractal temporal correlation might manifest the adoption of saddle-type and spiral-type CoP control, respectively. This understanding elaborates prior understandings that fractal temporal correlations reflect the coordination of corrections to sway across time (Blaszczyk and Klonowski, 2001; Amoud et al., 2007; Kuznetsov et al., 2013). Narrowing the angle between major and minor axes corresponds to fractal temporal correlations spread more homogeneously across angular directions. This 2D homogeneity reflects an opening of postural control to coordinate information across various potential task orientations. Conversely, increasing the angle between the two components of temporal correlations in CoP fluctuations might indicate a postural synergy collapsing the potential two-dimensionality of CoP into a 1D axis of relatively stronger temporal correlations. This may signify a more efficient use of sensorimotor integration for postural control canalized into a task-relevant orientation (e.g., Balasubramaniam et al., 2000). Subsequent investigations could test this hypothesis more explicitly using manipulations demanding releasing bodily degrees of freedom. Future work could also model the spatial distribution of temporal correlations from a holistic coordination perspective (Latash et al., 2007; Latash, 2012; Latash, 2024; Mangalam et al., 2020a; Mangalam et al., 2020b), considering the intricacies of individuals’ musculoskeletal features, training regimens, and even within the cohort of injured gymnasts.

The strength of the head-turning relative to closing eyes contrasts starkly with traditional attributions of minor role to vestibular rather than proprioceptive and visual information (Fitzpatrick and McCloskey, 1994; Peterka and Benolken, 1995; Ivanenko et al., 1999; Horlings et al., 2009; Wiesmeier et al., 2015). However, vestibular input plays a significant role in tonically activating anti-gravitative muscles through the lateral and medial vestibulospinal tracts, in conjunction with reticulospinal pathways (Fetter et al., 1991; Wilson, 2013). Unilateral suppression of vestibular processing by the postural syndrome observed in various animal species following unilateral vestibular system suppression (Lacour and Borel, 1993; Dieringer, 1995) and in humans as well (Curthoys and Halmagyi, 1995; Lacour et al., 1997; Redfern et al., 2004; Cousins et al., 2013). In the present case of gymnasts, angular nuances in CoP available from OFSCA might reflect sport-specific sensorimotor adaptations, encompassing lower extremity strength/power, proprioceptive acuity, and integrating vestibular information (e.g., Glass and Ross, 2021). The adequacy of sensory information crucial for maintaining balance and postural stability relies heavily on head stability during body movement and suprapostural tasks (Kelty-Stephen et al., 2021b; Mangalam et al., 2021). The so-called “Head-Stabilization Strategy (HSS)” is posited to mitigate potential ambiguities in interpreting sensory inputs for balance, primarily relying on a geocentric sensorimotor frame of reference (orientation to the vertical). However, incorporating egocentric (head orientation with respect to the body) or exocentric (orientation to environmental objects) frames of reference refines head stabilization (Lacour et al., 1997; Cullen, 2012). Individuals naturally leaning on the HSS-type geocentric frame of reference may diminish the angle between the two components due to uncertainties in interpreting vestibular information from head rotation. This entails proprioceptive reintegration by extending bodily degrees of freedom and leaning more into the 2D spiral-type distribution of more uniform temporal correlations across the 2D support surface.

Of course, within this topological question of saddle vs. spiral, the differences among angle or CoP or AP are less material than the implication of *H* as a control parameter on phase-plane axes. We intend that replacement explicitly as a substantive way to test the long-discussed proposal that fractal temporal correlations have something to do with control—with our term “something” intentionally vague to reflect the ambiguity of these proposals (Doyle et al., 2004; Pascolo et al., 2006; Amoud et al., 2007; Lin et al., 2008; Kirchner et al., 2012; Munafo et al., 2016; Ducharme and van Emmerik, 2018; Gilfriche et al., 2018; Quijoux et al., 2021). It is intriguing that Asai et al. (2009) offers control models to explain the proposed outcome of fractal temporal correlations in postural observables, assuming simplicity that fractal temporal correlations are sooner the product of the real underlying control parameters. However, just as topology is flexible regarding the specific labeling of axes, modeling is just a logical correspondence from premise to outcome. Without an experimental manipulation of the proposed outcome, the topological models generating fractal patterning from an angular position do not yet prohibit the converse possibility that fractal patterning has a non-zero causal status in influencing the angular change. We suspect that the fractal scaling variation may be a control parameter sooner. Hence, we intend the novel step of reversing the role of fractality from outcome to potential control parameter and, so, of testing the capacity of the saddle and spiral topologies to hold on a phase plane written in terms of *H* on the two dimensions of the force platform.

Given all of this interchangeability of parameters, it was for good reason that Weinberg coined his Third Law of Progress in Theoretical Physics: “You may use any degrees of freedom you like to describe a physical system, but if you use the wrong ones, you’ll be sorry” (Weinberg, 1983, p. 16). The reason for OFSCA is that the axes implicit in the observables forming the vector basis of the measurement could be wrong degrees of freedom, and flows within those bases might follow their intrinsic dimensionality. The power of the OFSCA method may be that it can take a fixed coordinate system like the 2D force platform, and it can then model the phase of anisotropic flow within that 2D coordinate system without using degrees of freedom with the wrong angular orientation to characterize that flow. *H* may be a suitable degree of freedom to embody control. Indeed, though we do not presume to manipulate *H* in this study, if *H* is a suitable candidate for postural control parameters, we should expect that *H* should embody the same abstract topological structures invoked in modern control theory. Any failure, at least, to find traces of known topology associated with intermittent control should hopefully be a strong point of evidence against rhetoric around fractal-themed control.

There is much yet to learn about the application of OFSCA to understanding postural control dynamics. At the very least, in the short term, we can say that the conventional premise, attributing postural control to the anatomical AP and ML axes, is incomplete. Unlike traditional stabilographic analysis, the OFSCA method unravels a potential disparity between this portrayed and actual control dynamics. In the longer term, we see a potential for OFSCA to inform theories of intermittent control using feedback and instability to reset postural synergies. Individuals adeptly tailor their posture control to align with task demands and anatomical constraints, exhibiting control in oblique directions to the AP and ML axes, complete with suborthogonal angles between the controlled axes. Furthermore, this spatial anisotropy in temporal correlations in CoP fluctuations diverges across individuals, potentially encapsulating variations in muscular strength, neurological control, and task-specific constraints. In contrast, the conventional orthogonal decomposition of the CoP trajectory adheres to a generic concept, lacking the discriminative power to distinguish among individuals in these critical dimensions. Closing the eyes diminished the angle between these two orientations, implying an amplified dependence on proprioception for postural control, effectively releasing bodily degrees of freedom across the 2D support surface. Subsequent head rotations accentuated this angular reduction, signifying a heightened liberation of bodily movement degrees of freedom. This process facilitates the integration of vestibular information with proprioceptive input, enhancing the overall control of posture. In contrast, head rotations failed to induce any discernible adaptation, with the posture swiftly reverting to its normal state within a single trial. This nuanced exploration provides valuable insights into the adaptive mechanisms gymnasts use to navigate sensory inputs during postural control.

Interestingly, the relationship between the standard deviation of temporal correlations (*SD*_*H*_) across the 2D support surface and the range of temporal correlations (Δ*θ*) is inversely proportional: larger *SD*_*H*_ coincides with larger Δ*θ*, while greater range is associated with smaller Δ*θ*. This suggests that extremal temporal correlations operate in the opposite direction from the overall standard deviation of temporal correlations. Visualizing this relationship as a bell-shaped curve, larger Δ*θ* corresponds to a broader distribution with shorter tails, while lower Δ*θ* corresponds to a narrower distribution with longer tails. Postural control maintains a balance in the spatial distribution of fractal temporal correlations, ranging between extremes and average variability. Future studies could delve deeper into this phenomenon, exploring its nuances in healthy adults, aging and diseased populations, and specifically trained groups such as gymnasts to provide a comprehensive understanding of the underlying mechanisms across diverse demographics.

The present treatment of gymnasts’ postural control dynamics offers a valuable gold standard for understanding what the OFSCA results might look like in one of the most dexterous populations. The targeted response of Δ*θ* to the head-turn and the prompt rebound in trained gymnasts makes the spiral-type angular homogeneity seem like an adaptive strategy. This case helps to clarify an earlier ambiguity as to how we might extend this OFSCA modeling strategy to understand the pathologies of motor control. For instance, Parkinson’s disease contributes to deficits in postural control, potentially disrupting directional stability and heightening susceptibility to falls (Bloem et al., 2001; Grimbergen et al., 2004). In comparing healthy adults and adults with Parkinson’s disease, we found that task-settings-inducing instability prompted the narrowing of Δ*θ* in healthy populations (Mangalam et al., 2024b). This finding aligns neatly with the present findings in trained gymnasts. We also found that even healthy older adults showed age-related and task-dependent narrowing. However, people with Parkinson’s showed narrowing of Δ*θ* that was less sensitive to task setting and more sensitive to their diagnosis. The present work represents our attempt to understand how gymnasts’ task-sensitive narrowing of Δ*θ* reflects known themes in the intermittent postural control strategy. This alignment of narrower and wider Δ*θ* with spiral-type and saddle-type control strategies offers the novel understanding that postural control with Parkinson’s disease may use the spiral-type strategy in excess—just as our head-turn manipulation signifies the impact of vestibular perturbation, it may be that the narrowing of Δ*θ* with age is a function of vestibular degeneration (Iwasaki and Yamasoba, 2015; Anson and Jeka, 2016) contributing to fall risk in the elderly (Liston et al., 2014). This minimal vestibular degeneration leaves intact the capacity for the postural control to use spiral-type release of postural degrees of freedom as a temporary response to perturbation. Adults with Parkinson’s may constitute an extreme case of spiral-type control, not necessarily from vestibular degeneration but from multifarious deficits in sensory integration rather than task-sensitive adaptation. Their more drastic and less task-sensitive reduction of Δ*θ* may thus reflect postural control that has lost the capacity to collapse degrees of freedom into synergies aligning with saddle-type modes. Hence, OFSCA offers an unprecedented capacity to dissociate multiple forms of change in fractal scaling, at once vindicating but crucially elaborating upon more straightforward notions of fractal temporal correlations as somehow crucial to health (Goldberger et al., 2002; Lipsitz, 2002; Torre et al., 2019). OFSCA opens up the possibility that more nuanced modeling of physiological anisotropy might make it possible to dissociate task-related changes in fractal temporal correlations from deficits due to more longitudinal or clinical concerns.

That being said, the present manuscript is not without its limitations. We have yet to ascertain the consistency of OFSCA results for an individual across repeated trials and whether differences in control patterns persist upon repeated testing, considering that subtle changes in task constraints could induce variation in control strategies. Furthermore, it remains to be investigated whether spatial variability in fractal temporal correlations can predict critical aspects of postural control, such as the ability to respond to unpredictable perturbations or resilience to falls during more dynamic scenarios such as walking. Expanding the application of OFSCA results to predict clinical performance measures would offer a novel and valuable contribution to our understanding of spatial variation in these fractal patterns. Spatial variation in fractal temporal structure has previously been linked to intermittent control strategies that regulate posture in healthy adults. Changes in this intermittency have been shown to elucidate posture in healthy older adults and older adults with Parkinson’s disease (Mangalam et al., 2024b), suggesting the potential of these findings to inform interventions to enhance postural stability across diverse populations. Future research involving high-performance athletes on one end of the spectrum and clinical populations on the other might provide complementary insights to establish the OFSCA results on a firmer theoretical foundation and elucidate the causal significance of fractal temporal correlations as a control parameter in postural adaptations.

On less theoretical grounds, an inherent limitation of our study is using only one trial per condition to assess postural control. Typically, postural control data are gathered across multiple trials to ensure reliability and consistency and to mitigate the impact of outlier trials. By employing only one trial per condition, the variability within each condition may not be fully captured, potentially limiting the generalizability of our findings. Furthermore, the duration of each trial, set at 20 s, may be considered relatively brief in the context of quiet-standing research. Extended trial durations are often preferred to stabilize postural adjustments and better capture nuanced changes in postural control over time. The short duration of our trials may restrict the depth of insight into participants’ postural control abilities and could affect the robustness of our conclusions. Future research endeavors should consider employing multiple trials per condition and extending the duration of each trial to enhance the comprehensiveness and reliability of postural control assessments.

Effective postural control amid intricate gymnastic tasks necessitates precise sensory integration and somaesthetic reweighting, as a failure in these can significantly heighten the risk of injury. The present study illuminates an unrecognized facet of gymnasts’ postural performance—spatial anisotropy in temporal correlations in CoP fluctuations, signifying postural control extending beyond the conventional anatomical AP and ML axes (Janusz et al., 2016). Anisotropy escapes most traditional portrayals of postural control following practical considerations like the output from force platforms, anatomical constraints, and inverted-pendulum modeling (Rocchi et al., 2004). The innovative OFSCA method affords empirical traction on postural control mechanisms long discussed in the literature Asai et al. (2009) but beyond the scope of these traditional portrayals. The OFSCA departure into modeling anisotropy explicitly has substantial implications, particularly in the realm of proprioceptive reintegration—a crucial aspect of sensorimotor integration that distinguishes gymnasts from their non-gymnast counterparts (Debu and Woollacott, 1988; Vuillerme et al., 2001a; Vuillerme and Nougier, 2004; Calavalle et al., 2008; Gautier et al., 2008; Busquets et al., 2021; Zemková and Kováčiková, 2023).

Potential physiological explanations for these findings may involve localized tissues or specific subsystems. Notably, heightened activity within the sympathetic nervous system has been associated with increased covariation among diverse physiological systems (Garcia et al., 2011). The robust connections between the visual and vestibular systems (Abekawa et al., 2018; Rühl et al., 2018) may elucidate these observed effects. However, the apparent simplicity of these explanations is quickly complicated by qualifications, interactions, and contextual sensitivities. For instance, the interactions between the visual and vestibular systems must be qualified by factors such as aging (Lui et al., 2019), migraines (Bednarczuk et al., 2019), neuritis (Roberts et al., 2018), and their interactions with somatosensation (Moro and Harris, 2018). Then again, what is specific needs not necessarily only to be what is local. These findings may be no less specific to physiological tissues that spread globally through the body and whose function is to mediate among various components at various scales. The present results are consonant with and supportive of explanations of perception-action that rest on the multiscaled “tensegrity”-like dynamics of connective-tissue networks that span the entire body (Turvey and Fonseca, 2014; Levin et al., 2017; Scarr et al., 2024). Here, the physiological substrates afforded by the fascia embody a complex geometrical relationship that balances various muscular, skeletal, and neural tissues. This specific anatomical relationship leverages abstract geometrical balances between tension and compression that proceed downward into the finest scales of cytoskeletal dynamics supporting cell motility (Ingber, 2006; Ingber, 2008) and upward to the larger scales of intentional organism coordination of behavior (Mangalam et al., 2020a; Mangalam et al., 2020b; Profeta and Turvey, 2018; Turvey and Fonseca, 2014; Van Orden et al., 2012). The nesting of tension-compression balances proceeds symmetrically across scales deformed only by anatomical and metabolic constraints. The result is a multifractal geometry reflecting a rich family of scale-invariant processes reflecting a common ancestry in the cascading dynamics of nonlinear cross-scale interactions. Indeed, nonlinear cross-scale interactions have become a major highlight of modern explanations for how the nervous system can provide such adaptive, context-sensitive support (Morgane et al., 2005; Thayer, 2006; Suckling et al., 2008; Changizi and Destefano, 2010; Deco et al., 2011; Damm et al., 2020; Stylianou et al., 2021).

The vast reach of connective tissue networks thwarts the simple reduction of our phenomena to local components. However, the support for such context-sensitive behavior is yet physiological. Support for the role of tensegrity-like connective tissue in perception and action appears through “ultrafast” adaptive responses unfolding faster than neural transmission could support, appearing within cells belonging to multicellular organisms (Ingber, 2006; Ingber, 2008), single-celled organisms (Grebecka et al., 1999), small organisms just aiming to stay upright (Frantsevich and Gorb, 2002; Endlein and Federle, 2013), as well as humans standing (Marsden et al. 1983), locomoting (Moritz and Farley, 2005; Kiely and Collins, 2016), and using language (Kelso et al., 1984; Moreno et al., 2011). Complicating matters for the physiologist aiming to reduce function to anatomical components, tensegrity may be more generic than the idiosyncrasies of individual model-organism anatomy. Although we can point here to likely vestibular contributions, various previously named model systems exhibit sensitivity to mechanical rotation without having any vestibular system (Brunet, 1951; Svidersky and Plotnikova, 2002; Dilão and Hauser, 2013). And that is not to say that everything can be a tensegrity system. On the contrary, tensegrity-based approaches to physiology offer the specific guarantee that the connective tissues serve to regulate a peculiar sort of geometry called “multifractal” (Turvey and Fonseca, 2014). The tensegrity mechanism’s specificity increases in abstract geometrical properties sooner than in anatomical materials. The physiological diversity offers many “anchors” that can enact generic geometrically invariant “templates” (Holmes et al., 2006).

Thus, the present work informs what Turvey and Fonseca (2014) called the “multifractal tensegrity” (MFT) hypothesis. The MFT hypothesis articulated two crucial points in light of the foregoing observations. First, it retrospectively proposed that the widespread evidence of multifractal fluctuations in physiological systems have inevitably entailed cascading tensegrity-like dynamics; this point was not exclusive to the MFT hypothesis but followed upon prior proposals that multifractal physiological fluctuations reflected cascade dynamics (Ivanov et al., 2001; West, 2006). The more forward-looking second point of the MFT hypothesis was that the empirically estimable multifractal profile of bodily coordination might provide an empirical means for modeling the control of dexterous action. This latter point has received extensive support through the correlational study of postural, suprapostural, perceptual, and cognitive performance (Bell et al., 2019; Booth et al., 2018; Carver et al., 2017; Dixon and Kelty-Stephen, 2012; Hajnal et al., 2018; Kelty-Stephen and Dixon, 2014; Kelty-Stephen et al., 2021b; Mangalam et al., 2020a; Mangalam et al., 2020b; Mangalam and Kelty-Stephen, 2021a; Mangalam et al., 2021; Stephen et al., 2012a; Teng et al., 2016) as well as through experimental tests of multifractal stimulation on some of the same performance measures (Stephen and Dixon, 2011; Ward and Kelty-Stephen, 2018; Bloomfield et al., 2021; Kelty-Stephen et al., 2023).

This prior work has shown a systematic relationship between prior multifractal stimulation or fluctuation and subsequent performance measures. There has thus been a major intermediary blank in the modeling where we might have only hoped that multifractality bore upon the topology of control strategies. The present work aims to bolster support for this second part of the MFT hypothesis by filling in that blank. Specifically, this work suggests that multifractality is not merely an epiphenomenon of some fundamentally non-multifractal control strategies but could be part and parcel of control itself. The present work provides initial positive evidence about the plausibility that multifractal parameters could themselves serve as control parameters governing topologies for postural control. It may be particularly compelling that we have not presumed any novel topologies for postural control here. It only invoked pre-existing topologies that had once been proposed to produce a multifractal structure. Postural control might reflect the interplay of yet-unfamiliar multifractal flows through well-established topological constraints.

The present proposal is that multifractal tensegrity acts on known topologies of postural control, and it calls for new elaborations of theoretical discourse. On the one hand, these results give new force to a long history of promises that fractal and multifractal fluctuations might support or govern postural control. These prior promises rested on circumstantial evidence of differences in fractal or multifractal outcomes between diagnosis groups or tasks (Lipsitz et al., 1990; Lipsitz and Goldberger, 1992; Duarte and Zatsiorsky, 2000; Blaszczyk and Klonowski, 2001; Lipsitz, 2002; Amoud et al., 2007; Kuznetsov et al., 2013). Although these works appeal to a role for endogenous stochastic processes exerting scale invariant form to postural corrections, perhaps from finding no better alternative at hand, they found this role for control in the exogenous dimensional frame of the orthogonal AP and ML axes of the force plate. And for all this commitment to the exogenous force-plate dimensionality, these proposals never made clear contact with the topologies that contemporary models of control have founded in force-plated dimensions and used to reduce fractality to inverted-pendulum dynamics (Bottaro et al., 2005; 2008; Asai et al., 2009; Asai et al., 2013; Ahn and Hogan, 2012; Nomura et al., 2013; Nakamura et al., 2021). On the other hand, the present work substantiates the rhetoric about fractal-based control by offering a more concrete foundation in the same topologies the inverted-pendulum models invoke. The major changes we propose here are twofold: first, thanks to the OFSCA modeling, we have identified the control topologies in a dimensional frame endogenous to the body rather than in the exogenous force-plate dimensions, and second, multifractality (i.e., literally, the variability in fractality) is the control-parametric domain on which the topology is defined rather than an interesting by-product.

On the other hand, the present work raises new questions about the tuning of control. A question for future research may be how multifractality enters the control process. For instance, various theories of postural control currently debate the relatively continuous or intermittent form of control (Lipsitz et al., 1990; Lipsitz and Goldberger, 1992; Duarte and Zatsiorsky, 2000; Blaszczyk and Klonowski, 2001; Lipsitz, 2002; Amoud et al., 2007; Kuznetsov et al., 2013). However, the longstanding premise for many of these theories is that the nervous system uses some form of feedback to enact control relative to an internal model. For instance, the longstanding appeal to internal models in motor control has invoked a drama played out between a representation of current bodily position and “efference copies” that encode intended changes to that position (Mittelstaedt, 1998; Kuo, 2005; Maurer et al., 2006). This drama implies that the nervous system explicitly tunes control toward correcting the error, narrowing the distance between the current and the desired posture. An important future question in continuation of the present work is thus whether our implication of multifractality as a control parameter explicitly means that the central nervous system “sets,” “monitors” and “regulates” multifractality. We suspect that answer is “no” for at least two reasons: the ergodicity-breaking behavior of biological activity thwarts representativity of the entire behavior by any single or sequence of observations (Stephen and Dixon, 2011; Mangalam and Kelty-Stephen, 2021b; Kelty-Stephen and Mangalam, 2023; Mangalam et al., 2023), and the multifractality we find in human movement control reflects both responsivities at multiple time scales and nonlinear convolution of responsivities across those scales (Mandelbrot, 1982; Stephen and Dixon, 2011; Lovejoy and Schertzer, 2018; Kelty-Stephen and Mangalam, 2022), ensuring stochastic casual support rather than the simple deterministic cause. We expect that the multifractality appearing in postural control is the consequence of multiplicative cascades (Furmanek et al., 2021; Kelty-Stephen, 2018; Kelty-Stephen et al., 2021b; Kelty-Stephen et al., 2021a; Mangalam and Kelty-Stephen, 2021a; Mangalam et al., 2021). We also expect that control topologies are sooner the emergent compromise between endogenous multifractal-dimensional cascade dynamics and the exogenous dimensionality of the force plate’s task constraints (Jacobson et al., 2021). However, these points will require more explicit tests than current evidence provides.

## Data Availability

The raw data supporting the conclusion of this article will be made available by the authors, without undue reservation.
